# Effectiveness of Fuyuan Xingnao Decoction for patients with diabetes mellitus combined cerebral infarction

**DOI:** 10.1097/MD.0000000000017273

**Published:** 2019-09-27

**Authors:** Chao Jiang, Ting Wang, Zhen Ma, Bang-jiang Fang

**Affiliations:** a The Third Department of Neurology, The Second Affiliated Hospital of Xi’an Medical University; b Department of Emergency, Longhua Hospital Shanghai University of Traditional Chinese Medicine, Shanghai; c School of Economics & Management, Xi Dian University; d Department of Cardiology, Xi’an Traditional Chinese Medicine Hospital, Xi’an, China.

**Keywords:** cerebral infarction, diabetes mellitus, effectiveness, Fuyuan Xingnao Decoction, randomized controlled trial, safety

## Abstract

**Background:**

Previous study has reported that Fuyuan Xingnao Decoction (FYXND) can be utilized for the treatment of patients with diabetes mellitus (DM) combined cerebral infarction (CI) effectively.

**Methods:**

We will search from the following databases of MEDLINE, EMBASE, Cochrane Library, PsycINFO, Global Health, Web of Science, Allied and Complementary Medicine Database, Chinese Biomedical Literature Database, and China National Knowledge Infrastructure. All databases will be searched from the inception to the present without language limitation. Two independent authors will perform literature selection, information collection, and methodological quality assessment. Statistical analysis will be carried out using RevMan 5.3 software.

**Results:**

This study will provide accurate results on the effectiveness and safety of FYXND on DM and CI through primary and secondary outcomes. The primary outcome is neurological deficit. The secondary outcomes consist of fasting blood glucose, hemoglobin Alc, fasting insulin, quality of life, and adverse effects.

**Conclusions:**

This well-designed study will establish high quality evidence of the effectiveness and safety of FYXND for DM and CI to facilitate the clinical practice and guideline development.

## 1 Introduction

Diabetes mellitus (DM) is a metabolic disorder and an independent risk factor of first stroke, and also an important factor for cerebral infarction (CI).^[1–3]^ It is characterized by elevated blood glucose and has been a public health problem.^[4–6]^ It has been estimated that the incidence in patients with DM is 2.5 to 3.5 times higher than patients with non-DM.^[7,8]^ In addition, about 20% of patients with CI have DM.^[8]^

There are several managements for patients with DM and CI.^[9–20]^ These interventions include mecobalamin, α-Lipoic acid, and Chinese herbal medicine (such as Fuyuan Xingnao Decoction [FYXND], Huoxue Jiangtang Decoction).^[9–20]^ Of those, FYXND is widely used for the treatment of patients with DM and CI in China.^[21–27]^ However, its efficacy is still inconclusive. Therefore, this study will firstly assess the efficacy and safety of FYXND for the treatment of patients with DM and CI.

## 2 Methods

### 2.1 Criteria for including studies

#### 2.1.1 Types of studies

All randomized controlled trials (RCTs) of FYXND for treating patients with DM and CI will be considered for inclusion. However, any other studies, such as animal studies, non-clinical studies, non-controlled studies, and quasi-RCTs will all be excluded.

#### 2.1.2 Types of interventions

The patients in the experimental group must receive FYXND.

The patients in the control group can receive any treatments, except any forms of FYXND.

#### 2.1.3 Types of patients

Participants diagnosed with DM combined CI will be included without any limitations of race, sex, and age.

#### 2.1.4 Types of outcome measurements

The primary outcome is neurological deficit, as measured by National Institutes of Health Stroke Scale or other scales. The secondary outcomes consist of fasting blood glucose; hemoglobin Alc; fasting insulin; quality of life, as assessed by activities of daily living; and adverse effects.

### 2.2 Search strategy

A systematic search of the following databases from the inception to the present without language limitation to identify relevant studies: MEDLINE, EMBASE, Cochrane Library, PsycINFO, Global Health, Web of Science, Allied and Complementary Medicine Database, Chinese Biomedical Literature Database, and China National Knowledge Infrastructure. The search strategy from MEDLINE is presented in Table 1. Any similar search strategies for other electronic databases will be adapted.

**Table 1:**
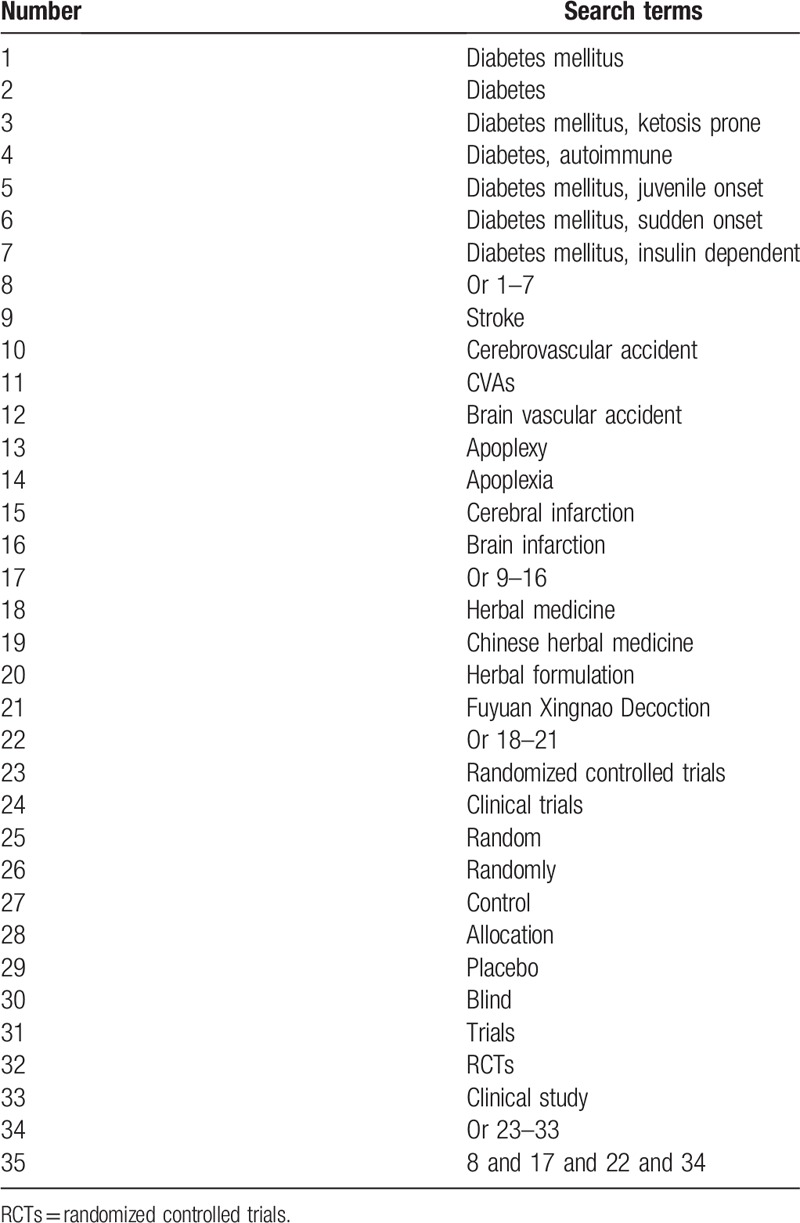
Search strategy utilized for MEDLINE database.

Additionally, we will also search clinical registry, conference proceedings, and reference lists of included studies.

### 2.3 Data collection and analysis

#### 2.3.1 Study selection

Two independent authors will scan the titles and abstracts yielded from the electronic databases searches against the pre-designed inclusion criteria. If the titles and abstracts indicate that literature is uncertainty, the full-text records will be read further to help decide. If there is still some insufficient information for a decision to be made about eligibility, we will inquire additional information from the primary study authors. All excluded studies with record reasons will be presented. Any disagreements will be resolved by mutual discussion with the help of a third author. The process of study selection will be showed in flowchart.

#### 2.3.2 Data extraction

Two independent authors will collect data from all eligible studies. Any discrepancy will be settled down by a third author through discussions or consultations with a third author. We will collect following information: study characteristics, including title, first author, year of publication, location, language, eligibility criteria, etc; patient characteristics, including sex, age, comorbidities, sample size, etc; study setting; study methods, including randomization, blinding, concealment, etc; treatment details; outcome measurements, safety, conflict of interests, and others.

#### 2.3.3 Missing data dealing with

Where the data is unclear, insufficient, or missing, original authors of primary eligible studies will be contacted to require those data. If we cannot achieve that data, only available data will be analyzed and will be discussed in the text.

#### 2.3.4 Risk of bias assessment

We will utilize the Cochrane risk of bias tool for the methodological quality assessment for each eligible study. Seven aspects of this tool will be evaluated, and each item will be further assessed as low, unclear, and high risk of bias. Two independent authors will perform risk of bias assessment, and any different opinions will be solved through discussion with a third senior author.

#### 2.3.5 Methods of treatment measurements

We will apply risk ratio and 95% confidence intervals for dichotomous data, and mean difference or standardized mean difference and 95% confidence intervals for continuous data.

#### 2.3.6 Assessment of heterogeneity

*I*^2^ statistics will be used for identifying heterogeneity for each outcome among eligible studies. *I*^2^ ≤ 50% indicates acceptable heterogeneity, while *I*^2^ > 50% means significant heterogeneity.

#### 2.3.7 Assessment of reporting bias

We will assess the possibility of reporting bias using funnel plot and Egger regression test if >10 eligible studies are included.^[28,29]^

### 2.4 Data synthesis

We will use RevMan 5.3 software for statistical analysis. If the heterogeneity is acceptable (*I*^2^ ≤ 50%), a fixed-effect model will be used for data synthesis, and meta-analysis will be carried out. On the other hand, if the heterogeneity is significant (*I*^2^ > 50%), a random-effect model will be utilized for data synthesis, and subgroup analysis will be performance. If there is still significant heterogeneity after subgroup, a narrative description of the outcome results will be reported instead of meta-analysis.

#### 2.4.1 Subgroup analysis

We will perform subgroup analysis and meta-regression to identify any sources of substantial heterogeneity if necessary according to different types of treatments, comparators, and outcome measurements.

#### 2.4.2 Sensitivity analysis

We will investigate the robustness of pooled results by removing studies with high risk of bias.

## 3 Discussion

Numerous studies have reported that FYXND can be used for the treatment of patients with DM and CI effectively. However, no study has systematically assessed the efficacy and safety of FYXND for DM and CI. The results of this study will summarize the latest evidence of FYXND for treating patients with DM and CI. This study will also provide helpful evidence and research directions for further researchers and clinicians.

## Author contributions

**Conceptualization:** Chao Jiang, Ting Wang, Zhen Ma, Bang-jiang Fang.

**Data curation:** Ting Wang, Bang-jiang Fang.

**Formal analysis:** Chao Jiang, Ting Wang, Zhen Ma.

**Funding acquisition:** Bang-jiang Fang.

**Investigation:** Bang-jiang Fang.

**Methodology:** Ting Wang, Zhen Ma.

**Project administration:** Bang-jiang Fang.

**Resources:** Chao Jiang, Ting Wang, Zhen Ma.

**Software:** Chao Jiang, Ting Wang, Zhen Ma.

**Supervision:** Bang-jiang Fang.

**Validation:** Chao Jiang, Ting Wang, Zhen Ma, Bang-jiang Fang.

**Visualization:** Chao Jiang, Ting Wang, Zhen Ma, Bang-jiang Fang.

**Writing – original draft:** Chao Jiang, Ting Wang, Zhen Ma, Bang-jiang Fang.

**Writing – review & editing:** Chao Jiang, Ting Wang, Zhen Ma, Bang-jiang Fang.
