# Effectiveness of Fuyuan Xingnao Decoction for patients with diabetes mellitus combined cerebral infarction: Erratum

**DOI:** 10.1097/MD.0000000000017886

**Published:** 2019-11-01

In the article, “Effectiveness of Fuyuan Xingnao Decoction for patients with diabetes mellitus combined cerebral infarction”,^[1]^ which appeared in Volume 98, Issue 39 of *Medicine*, the institute information of Zhen Ma is Xi’an Traditional Chinese Medicine Hospital. However, the correct information should be Xi’an Hospital of Traditional Chinese Medicine.
